# Replication-Associated Recombinational Repair: Lessons from Budding Yeast

**DOI:** 10.3390/genes7080048

**Published:** 2016-08-17

**Authors:** Jacob N. Bonner, Xiaolan Zhao

**Affiliations:** 1 Molecular Biology Program, Memorial Sloan Kettering Cancer Center, New York, NY 10065, USA; jab2050@med.cornell.edu; 2 Programs in Biochemistry, Cell, and Molecular Biology, Weill Cornell Graduate School of Medical Sciences, New York, NY 10065, USA

**Keywords:** recombination intermediates, template switch, HJ resolution, replication fork regression, SUMOylation, ubiquitination

## Abstract

Recombinational repair processes multiple types of DNA lesions. Though best understood in the repair of DNA breaks, recombinational repair is intimately linked to other situations encountered during replication. As DNA strands are decorated with many types of blocks that impede the replication machinery, a great number of genomic regions cannot be duplicated without the help of recombinational repair. This replication-associated recombinational repair employs both the core recombination proteins used for DNA break repair and the specialized factors that couple replication with repair. Studies from multiple organisms have provided insights into the roles of these specialized factors, with the findings in budding yeast being advanced through use of powerful genetics and methods for detecting DNA replication and repair intermediates. In this review, we summarize recent progress made in this organism, ranging from our understanding of the classical template switch mechanisms to gap filling and replication fork regression pathways. As many of the protein factors and biological principles uncovered in budding yeast are conserved in higher eukaryotes, these findings are crucial for stimulating studies in more complex organisms.

## 1. Introduction

Faithful genome duplication requires complete and accurate copying of the genome once per cell cycle. This process is frequently challenged by many types of replication impediments, such as tightly-bound non-histone proteins, intrinsically difficult to replicate genomic loci and DNA lesions generated from both intrinsic and exogenous sources [1]. Failure to properly manage these obstacles or to rescue impaired replication forks can lead to replication fork stalling and collapse and, consequently, genome instability and chromosomal rearrangements [2,3]. As these consequences underlie many human diseases, such as cancer, understanding the mechanisms that prevent them from occurring is fundamentally important for human health [1,4].

**Figure 1:**
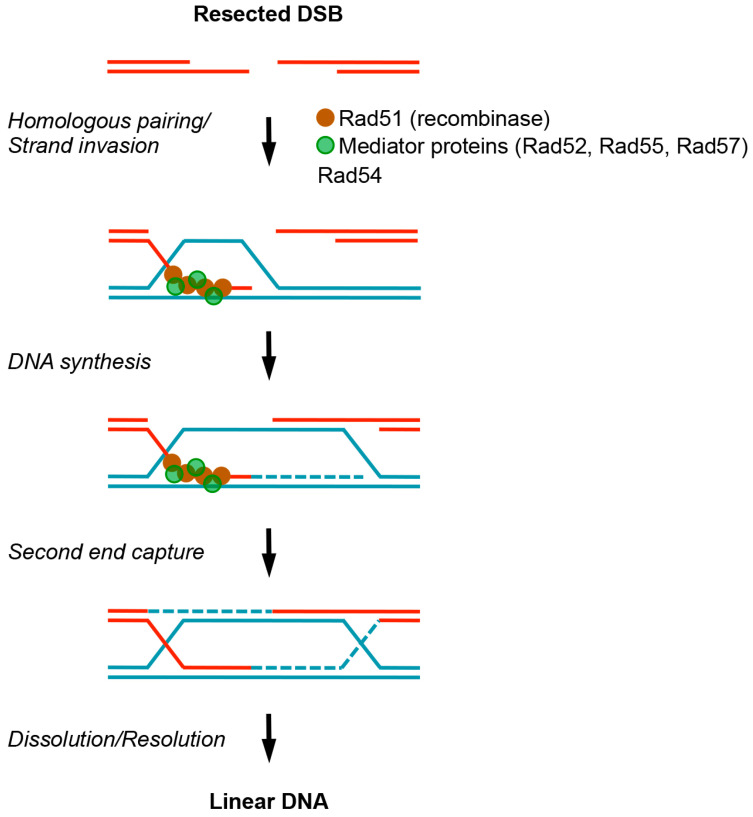
Simplified schematic of homologous recombination repairing DNA breaks. Recombinational repair of DNA double-strand breaks (DSBs) is initiated by resection away from the break, generating ssDNA. Rad51 then forms filaments along ssDNA with the help of mediator proteins, and then together with additional factors (including Rad54), they facilitate the Rad51-ssDNA filament in homology search and strand invasion into homologous sequences. This leads to the formation of DNA D-loop structures. After DNA synthesis of the invading strand, second end capture generates double Holliday junctions, which can then be processed by dissolution and resolution pathways, as described in the text. For further details, see [6,7].

While many mechanisms are involved in overcoming replication blockage [5], recombinational repair is one of the most important. Similar to the repair of DNA double-strand breaks (DSBs), recombinational repair that aids replication requires the core set of recombination proteins. In budding yeast, these include the Rad51 recombinase and mediator proteins, such as Rad52, Rad55, and Rad57, that facilitate the formation of Rad51-ssDNA filaments, as well as proteins, such as Rad54, that facilitate strand invasion and homologous pairing [6]. Subsequently, repair synthesis leads to the formation of newly-synthesized DNA and recombination intermediates, such as single and double Holliday junctions (HJs), which are processed into linear DNA molecules by enzymes, such as the Mus81-Mms4 complex and the Sgs1-Top3-Rmi1 (STR) complex. Detailed understanding of how core recombination proteins catalyze common recombination steps has been summarized in recent reviews [6,7]; thus, we only provide a schematic (Figure 1) to outline these steps, without further elaboration.

**Figure 2:**
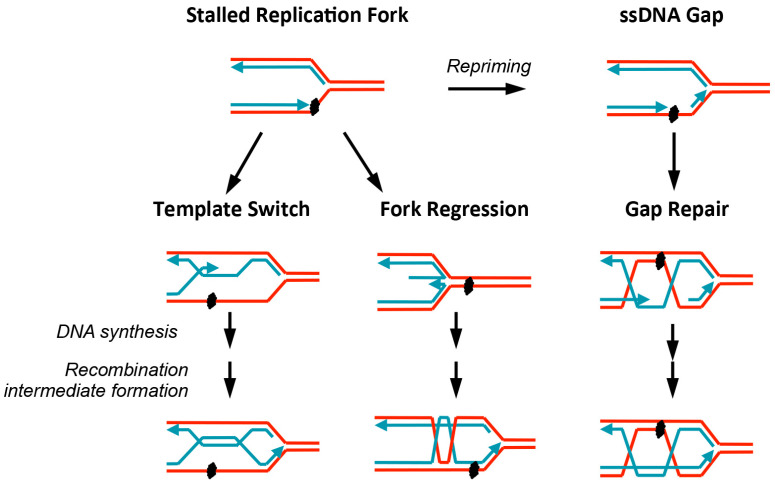
Replication-associated recombinational repair processes. Upon encountering DNA template lesions, replication forks stall and can be repaired by multiple modes of recombinational repair. The template switch pathway (left) uses the homologous sequence on the sister chromatid as a template to replicate over the damaged region. The fork regression pathway (middle) entails the dissociation of nascent strands from their templates and their annealing with each other. One of the nascent strands can utilize the other as a template for limited DNA synthesis and/or invade the homologous template strand, forming recombination intermediates. In other situations, replication may resume downstream of a template lesion, leaving behind an ssDNA gap (right). Such a gap can be filled through recombination using the sister chromatid as a template. All three situations depicted generate DNA intermediates in the form of joint molecules, which must be processed through dissolution or resolution. It is of note that only one possible model for each situation is shown here, though others are also feasible.

**Figure 3:**
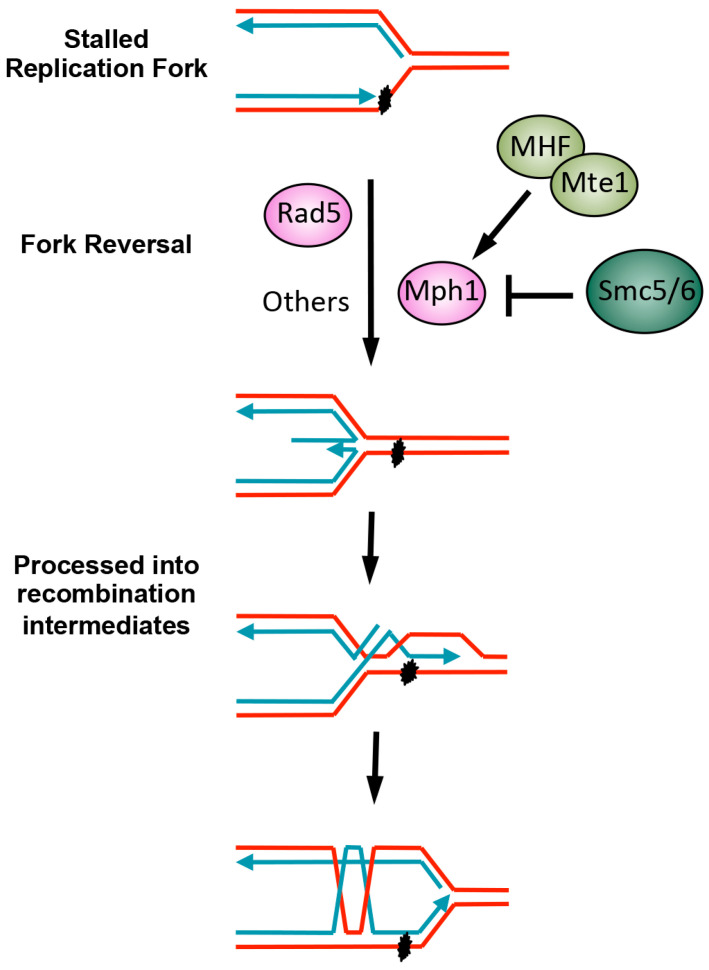
Schematic of replication fork regression and its regulation in yeast. Stalled replication forks can be reversed through the action of DNA helicases, such as Rad5, Mph1, and others, as described in the text. The regressed fork can be processed further, for example undergoing end resection or DNA synthesis. One possible outcome entails the invasion of homologous template strands, leading to the generation of recombination intermediates in the forms of DNA joint molecules, which will need to be processed before chromosome segregation. The role of Mph1 in fork regression is inhibited by the Smc5/6 complex, and MHF and associated Mte1 help alleviate this effect.

This review focuses on the significant distinction between replication-associated repair and DSB repair, that is the involvement of factors coupling the diverse stalled replication situations to different forms of recombinational repair. In particular, when replication forks encounter template lesions, at least three forms of recombination processes can take place (Figure 2). First, the template switch process can rescue stalled replication. In this mechanism, the newly-synthesized sister chromatids, which are identical or highly homologous to the original templates, can be used to make new copies of DNA [8]. To initiate this process, proteins that generate and read various modifications of the polymerase clamp PCNA are critical, due to their roles in signaling to the recombinational factors that catalyze the template switch [8]. Second, stalled replication forks can regress and subsequently engage in recombination. In this process, nascent DNA strands dissociate from their templates and anneal with one another to form a partial duplex, allowing the template strands to re-anneal [9]. After limited DNA synthesis and processing, one of the regressed strands can invade and pair with the homologous template strand to form recombination intermediates and re-establish replication forks (Figure 3). To initiate this process, the DNA helicase and motor proteins that catalyze replication fork regression (referred to simply as fork regression) are critical for coupling stalled forks with recombinational repair [9]. Third, repriming may occur downstream of the stalled replication forks, generating ssDNA gaps, which can then be repaired with the help of core and specialized recombination factors [7]. We describe each of these processes in more detail in the sections below.

**Figure 4:**
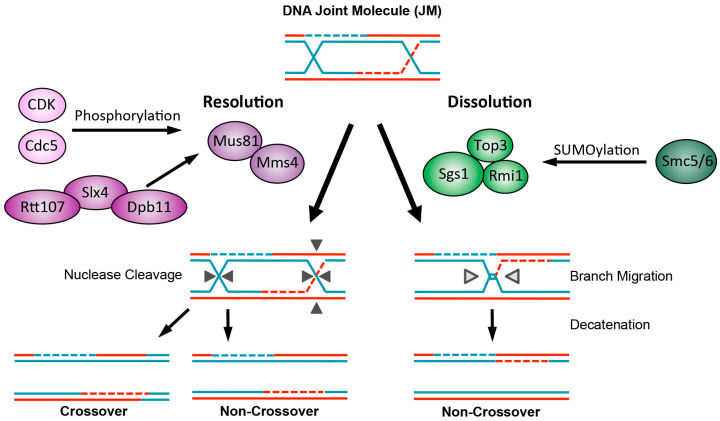
Two pathways for DNA joint molecule (JM) processing are regulated by phosphorylation and SUMOylation. Recombinational repair produces intermediates in the forms of JMs, such as double Holliday junctions, which are processed by two mechanisms: dissolution and resolution. In JM dissolution, the combined branch migration and decatenation activities of the Sgs1-Top3-Rmi1 complex process JMs into non-crossover products. The function of Sgs1-Top3-Rmi1 in this process is promoted by Smc5/6-mediated SUMOylation, likely in the S and G2 phases. In JM resolution, several nucleases, such as Mus81-Mms4, directly cleave JMs, resulting in a mixture of crossover and non-crossover products. Phosphorylation of Mms4 by CDK and Cdc5, in conjunction with the Slx4-Dpb11-Rtt107 scaffold complex, promotes Mus81-Mms4 activity in mitosis.

In addition to proteins that couple stalled replication with repair, replication-associated recombination is also greatly influenced by chromatin factors and cell cycle regulators. For example, as sister chromatids provide the most faithful template for repair, factors that influence cohesion and other aspects of chromatin states also contribute to replication-coupled recombinational repair [10]. The processing of recombination intermediates also relies on factors that modulate DNA cleavage enzyme functions and link them to distinct phases of the cell cycle (Figure 4) [11].

Many studies in recent years have yielded insights into the proteins especially important during replication-associated recombinational repair. Much work has been done in cells replicating in the presence of DNA-damaging agents that increase the burden of template lesions, such as the alkylating agent methyl methanesulfonate (MMS). A great deal of progress has been gleaned from the study of model organisms, such as the budding yeast *Saccharomyces cerevisiae*, where multiple genetic and physical methods can be readily employed. While higher eukaryotic organisms are undoubtedly more complicated, many aspects of replication-coupled recombinational repair, including the general principles and protein factors involved, are conserved. This review summarizes recent progress made in budding yeast to elucidate these conserved aspects of the repair, with references to higher eukaryotic situations when applicable. 

## 2. PCNA Modifications and Their Link to Recombinational Repair

PCNA is a ring-shaped homotrimeric complex that encircles DNA and promotes DNA synthesis by increasing DNA polymerase processivity. Upon replication blockage, PCNA can be ubiquitinated through two distinct ubiquitin E2-E3 pairs. The Rad6 and Rad18 pair enables mono-ubiquitination on lysine 164 of PCNA, while the Rad5 and Ubc13-Mms2 pair extends this modification into a K63-linked poly-ubiquitin chain [12,13]. The latter event leads to template switching-based recombination mechanisms [14]. This is relatively mutation-free compared to the pathway mediated by translesion polymerases, which can interact with the mono-ubiquitinated form of PCNA [7]. Historically, the pathways governed by PCNA mono- and poly-ubiquitination have been termed error-prone translesion synthesis and error-free DNA damage tolerance, respectively, and are highly conserved. Further details regarding early studies of these two pathways have been well summarized previously, and we refer the reader to these articles for additional reading [7,15,16,17].

### 2.1. Readers of PCNA Modifications

A major unanswered question about the above pathways has been how exactly PCNA poly-ubiquitination can lead to recombinational repair. Thus far, only one reader of PCNA poly-ubiquitination, Mgs1, has been reported in yeast [18]. Mgs1 is a DNA-dependent ATPase with single strand annealing activity, and its human homolog, WRNIP1, is also implicated in replication fork restart [19,20,21]. Earlier studies showed that Mgs1 physically and genetically interacts with DNA polymerase delta subunits and is implicated in replication restart [21,22,23,24]. A recent study demonstrated that Mgs1 directly interacts with poly-ubiquitinated PCNA and disrupts PCNA association with polymerase delta [18]. This effect is thought to be helpful for Rad51-mediated strand invasion, as the polymerase has to be removed from the DNA end before strand invasion can occur [18]. However, as *mgs1Δ* cells, unlike those lacking PCNA poly-ubiquitination, are not sensitive to replication stress, redundant or alternative pathways must exist that can recognize the PCNA poly-ubiquitination signal. In this regard, the human ZRANB3 helicase has been shown to bind poly-ubiquitinated PCNA and lead to replication fork regression, an event that can be channeled into recombinational repair [25]. Though ZRANB3 is not conserved in yeast, a similar mechanism may be employed, as yeast also possesses DNA helicases capable of fork regression (see below). Whether these helicases can read the PCNA poly-ubiquitination signal will be interesting to examine.

PCNA can also be modified in other ways. In particular, its SUMOylation at K164 and K127 disfavors recombination by recruiting the anti-recombinase Srs2 to sites of stalled replication [12,26,27]. The Srs2 helicase contains a SUMO-interacting motif (SIM) next to its PCNA interacting protein box (PIP box), and these two motifs synergistically promote its association with SUMOylated PCNA [28,29]. The resulting interaction is thought to disfavor Rad51 association near stalled replication forks [26,27,30,31]. A recent study further suggests that Srs2 can also inhibit DNA repair synthesis during recombination independently of its impact on Rad51 [32], indicating multi-pronged effects of this helicase. 

### 2.2. Control of PCNA and Srs2 Levels at Stalled Forks

The studies summarized above show that PCNA modification states and readers of these states play important roles in choosing between pro- and anti-recombinogenic modes during replication. Additional factors influencing this choice are those that can modulate PCNA and Srs2 levels. Several studies have implicated Elg1, a subunit of an RFC-like complex, in unloading PCNA from chromatin. This is mediated by the interaction of Elg1 with PCNA through its PIP box and SIMs [33,34]. Though Elg1 can unload PCNA without SUMOylation, SUMO may increase the efficiency of this process [33,34]. Without Elg1, PCNA retention on chromatin increases, leading to higher levels of MMS sensitivity and genome instability [35]. Though the underlying mechanisms accounting for the observed genome instability have yet to be delineated, it is possible that accumulated PCNA can associate with its many interactors, such as Srs2, which would bias against recombination even when it is needed.

Srs2 itself is subjected to regulation. The SUMO-like domain-containing protein Esc2, which associates with stalled replication forks through its DNA binding ability, can interact with Srs2 through the Srs2 SIM [36]. Esc2 was suggested to promote the turnover of chromatin-bound Srs2, thus exerting a local control of Srs2 levels at stalled forks [36]. In principle, such a function favors template switching and the formation of recombination intermediates. It is of note that Esc2 has also been ascribed a role in the resolution of recombination intermediates [37,38], and it is unclear whether this is related to Srs2 or occurs through a separate mechanism. These new findings have begun to illuminate a complex regulation of the PCNA-Srs2 axis that modulates pro- and anti-recombinogenic processes during replication. Future challenges include generating an integrated view regarding how these competing mechanisms take place at specific fork stalling situations and the detailed manner by which each mechanism can benefit replication and genomic stability.

## 3. The Shu Complex Promotes Rad51 Function in Recombinational Repair

### 3.1. Genetic Studies of the Shu Complex

While the template switch pathway linked to PCNA poly-ubiquitination occurs at or near stalled replication forks as described above, many ssDNA gaps behind replication forks must be filled by recombinational repair (Figure 2) [39]. This latter process logically requires the core recombination proteins, but it also relies on additional specialized recombination factors. One such factor is thought to be the Shu complex, composed of Shu1, Shu2, and the Rad51 paralogs Csm2 and Psy3. Genetic screens originally identified these genes as having a role in recombinational repair and in reducing mutation rates, likely by disfavoring the use of translesion synthesis pathways in coping with damaged DNA templates [40,41,42]. Further examination of the genetic relationship between the Shu complex and factors involved in various steps of recombinational repair has provided a better understanding of its function. For example, Shu complex mutants suppress the replication stress sensitivity caused by the lack of the HJ dissolution complex Sgs1-Top3-Rmi1 (STR) [41,43]. Additionally, Shu complex mutants rescue the synthetic lethality between mutants of STR and a HJ resolution complex Mus81-Mms4 [43]. These observations suggest that the Shu complex plays a role in generating recombination intermediates that require STR and Mus81-Mms4 for processing. A study by Mankouri et al. provided physical evidence for this model, as their 2D gel analysis showed that the Shu complex is required for generating recombination structures when cells replicate in MMS [44]. As loss of the Shu complex also suppresses the MMS sensitivity of *rad54Δ* and the synthetic lethality between *rad54Δ* and *srs2Δ* [43], it likely acts at a step prior to Rad54 function, such as in the formation, maintenance, and/or remodeling of the Rad51-ssDNA nucleoprotein filament.

### 3.2. Mechanisms of Shu Complex Functions

Biochemical and structural studies in the past several years have provided a mechanistic understanding of how the Shu complex performs these functions. The structure of the Csm2-Psy3 subcomplex mimics that of a Rad51 dimer, the functional unit of Rad51 nucleoprotein filaments [45,46,47]. In particular, the Csm2 and Psy3 interface is similar to the Rad51 dimer interface, and they share critical features at the regions where the Rad51 dimer interacts with DNA [45,46,47]. Even though Csm2 and Psy3 are devoid of ATP binding sites, unlike Rad51, these structural similarities suggest that they have the potential to interact with DNA in a similar fashion to Rad51 [45,47]. Indeed, the Csm2-Psy3 dimer binds various forms of DNA, such ssDNA, dsDNA, fork structures, and 3’overhang structures, without sequence preference [45,47,48]. Though Shu1 and Shu2 show no obvious DNA binding, they improve the association of the Shu complex with DNA [47]. As the Shu complex accumulates at DNA lesions independently of Rad51 and mediator proteins, its DNA binding ability may serve as a DNA-targeting mechanism; consistent with this notion, mutations affecting this feature cause MMS sensitivity [45,47].

In addition to directly interacting with DNA, Csm2 also binds to Rad55 and its partner Rad57, resulting in the indirect association of the Shu complex with Rad51 [48,49,50]. A recent study showed that these interactions support collaboration between the Shu complex, Rad55–Rad57, and Rad52 to promote Rad51 presynaptic filament assembly in vitro [49]. Such a role is consistent with the epistatic relationship between mutants of Rad55–Rad57 and the Shu complex [48,50,51]. As Csm2 mutants that affect its interaction with Rad55, but not Psy3 exhibited a null-like phenotype in terms of genotoxic sensitivity and the reduction of Rad51-mediated gene conversion, the Csm2-Rad55 interaction is an important aspect of Shu complex functions [49]. With that said, the Shu complex likely has additional roles. Shu2 was reported to be associated with Srs2 in both budding and fission yeasts [52,53]. Genetic studies have suggested that the Shu complex may directly or indirectly restrain Srs2 foci [54]. In addition, study of the *C. elegans* homolog of the Shu complex has suggested yet another role in remodeling the Rad51 nucleoprotein filament for more efficient strand exchange [55,56]. Determining whether these roles of the Shu complex and its homologs are conserved will help to generate a more comprehensive view of its functions.

The functions of the Shu complex described above could, in principle, affect all types of recombinational repair. However, in mitotic cells, the Shu complex is mainly required during S phase when replication is blocked by template lesions, but is dispensable for DSB repair [51]. These observations suggest that the Shu complex likely has a role in coupling replication to recombinational repair. A few suggestions for such a role have been made based on in vivo and in vitro studies. For example, the ability of the Shu complex to bind DNA and promote Rad51 filament formation suggests that it could help to repair ssDNA gaps behind replication forks or to help stabilize replication forks [45]. In addition, the Shu complex may work either independently of or concertedly with the PCNA modification pathways depending on the replication situations [37,57]. Testing of these ideas, such as examining the replication situations where the Shu complex can couple replication with recombination, will be important to gain mechanistic understanding of the role of this complex and its mammalian homologs, including several Rad51 paralogs, in replication.

## 4. Replication Fork Regression by DNA Helicases and Their Regulation

### 4.1. The Multiple Rad5 Activities

The Rad5 protein, described above for its role in promoting template switching, has functions besides ubiquitinating PCNA. It also possesses DNA helicase activity, which can lead to the regression of replication fork-like structures in vitro [58]. As the human homolog of Rad5, HLTF, also catalyzes PCNA poly-ubiquitination and fork regression, these proteins likely have conserved roles in replication [59]. Considering that both activities pertain to replication fork rescue, understanding their functional relationship is necessary for building an integrated view of how Rad5 and its homologs function.

A close relationship between the two Rad5 activities is intimated by its domain arrangement, wherein its ubiquitin ligase domain is embedded within its helicase domain. Two recent studies found that a mutation of the Walker B motif in the Rad5 helicase domain indeed reduces PCNA poly-ubiquitination [60,61]. However, since mutation of another helicase motif (motif VI, *rad5-QD*) sustains PCNA poly-ubiquitination, the helicase activity per se is not required for the modification [61]. Rather, the Walker B motif of the Rad5 helicase domain promotes PCNA interaction with the ubiquitin E2 enzyme Ubc13, an effect likely facilitating the nearby Rad5 E3 domain in ubiquitin transfer [60,61]. Thus, a Rad5 helicase motif plays a structural role in supporting its E3 function. As *rad5-QD*, which affects its helicase activity, but not PCNA modification, reduces resistance to MMS, but not levels of recombinational repair, the Rad5 helicase activity per se has functions independent of those mediated by PCNA poly-ubiquitination. The notion that two Rad5 activities make separate contributions to genotoxic resistance is also supported by other studies that examined Rad5 functions in the presence of alkylating agents [62,63]. Whether the Rad5 helicase function is solely to promote fork regression or is also involved in other DNA transactions that promote genotoxic resistance needs to be further examined. It is noteworthy that Rad5 can also promote translesion synthesis through a direct interaction with the factor Rev1 [64,65]. These three functions of Rad5 make it the most complex player within the post-replicative repair pathway.

### 4.2. Mph1 and Its Regulation

The budding yeast DNA helicase Mph1 and its homologs, including the human FANCM protein that is mutated in Fanconi anemia, also exhibit fork regression activities [66]. In yeast, lacking both Rad5 and Mph1 helicases is additive in causing genotoxic sensitivity [37], suggesting that they make separate contributions to fork regression and possibly other repair steps. Like Rad5, Mph1 is multi-functional: aside from catalyzing fork regression, Mph1 also promotes branch migration and D-loop dissociation in vitro and regulates crossover control in vivo [66]. These distinct roles for Mph1 intimate a need for differential regulation under specific circumstances. Mph1 regulatory mechanisms have emerged from several recent studies. One Mph1 regulator is the conserved Smc5/6 complex, an important genome maintenance complex whose mutations underlie chromosomal breakage syndromes [67,68,69]. Smc5/6 deficiency in budding yeast causes genotoxic sensitivity, accumulation of recombination intermediates, and lethality [70,71,72]. Interestingly, these defects are elevated by Mph1 helicase mutations or *MPH1* deletion [37,73,74]. Biochemical studies demonstrated that Smc5/6 directly binds to an Mph1 C-terminal regulatory region and restrains Mph1-mediated fork regression by preventing Mph1 oligomer assembly at fork junctions [73,74,75] (Figure 3). This interaction does not affect Mph1 D-loop dissociation functions or associated crossover control, indicating that Smc5/6 is a specific regulator of Mph1-mediated fork regression [75]. As disrupting Smc5/6 inhibition of Mph1 partially suppresses the MMS sensitivity of a Rad5 helicase mutant, hyper-active Mph1 may compensate for the Rad5 mutant’s fork regression defects [75]. More recent studies extended this regulatory circuitry by showing that Smc5/6 binding and inhibition of Mph1 are counterbalanced by the histone-fold complex MHF, composed of the conserved proteins Mhf1 and Mhf2 [76] (Figure 3). MHF appears to work with the Mte1 protein, which directly binds to DNA and helps MHF in promoting Mph1 functions [77,78,79]. The mechanisms of Mte1 function are not completely understood, though biochemical data suggest that it could give Mph1-MHF a preference for acting on certain DNA structures or directly stabilize D-loops. More recently, Mph1 phosphorylation was implicated in regulating mating-type switching [80], a form of DSB repair, though the implications of this modification on other Mph1 functions remain to be explored.

### 4.3. Other DNA Helicases Involved in Fork Regression and Additional Regulators

Fork regression appears to not be limited to Rad5 and Mph1, as the Rrm3 and Pif1 helicases also promote this process in vivo [81]. The functions of Rrm3 and Pif1 can be inhibited by the checkpoint kinase Rad53 that phosphorylates both helicases upon replication stress [81]. In the absence of Rad53, reversed and broken replication forks accumulate in a manner depending on both helicases, leading to poor recovery from replication stress [81]. Additional regulators of fork regression include DNA nucleases, Exo1 (in budding yeast) and Dna2 (in fission yeast), which prevent fork reversal presumably by resecting nascent strands [82,83]. In the case of Dna2, its phosphorylation by checkpoint kinases target it to stalled replication forks to disfavor fork regression [83]. Checkpoint signaling also discourages fork regression by reducing the topological stress caused by tethering ongoing transcripts to nuclear pore complexes: phosphorylation of several nucleoporins by Rad53 favors the release of the transcripts from nuclear pores, reducing the opportunity for topologically-induced fork regression [84].

In summary, current studies suggest that fork regression is both positively and negatively regulated. The complex regulation is likely because, while this process can lead to fork restart, it can also generate DNA breaks when regressed forks are cleaved or complex DNA structures that may be difficult for the cellular machinery to process in a timely manner. Multi-layered regulation as elaborated above may provide a window of opportunity for cells to utilize other mechanisms to restart replication, such as translesion synthesis or template switching, yet still maintain fork regression as a backup pathway or restrict its use to specific loci. A similar scenario is likely in mammals, wherein multiple enzymes with fork regression activity have been found, some of which are negatively regulated by checkpoint signaling [25,85,86]. Further studies will be needed to provide a deeper understanding into the mechanisms that dictate each mode of regulation described, as well as their integration with other fork rescue mechanisms.

## 5. Recombination Intermediate Processing Is Controlled by Protein Modifications

Recombination intermediates, also called DNA joint molecules (JMs), such as D-loops and HJs, must be resolved before chromosome segregation; otherwise, chromosomal nondisjunction occurs, leading to various forms of genome instability [87]. Cells have multiple mechanisms for processing JMs (Figure 4). First, a process termed dissolution entails the collective activities of the Sgs1 helicase, Top3 topoisomerase, and their cofactor Rmi1 (together STR) [88]. This mechanism generates relatively conservative non-crossover products. Endonucleolytic resolution is a second way for cells to process JMs, through cleavage by nucleases, such as Mus81-Mms4 and Yen1 [11]. Such nuclease-based mechanisms generate crossover products in addition to non-crossover products, with the former containing more genetic exchanges. In addition, these nucleases can act on replication structures, causing undesired cleavage. For these reasons, their use is restricted until G2/M to remove any JMs that remain unprocessed, while STR is preferred in most parts of the cell cycle. Consistent with this difference in the relative contribution to JM processing, the lack of STR leads to accumulation of JMs that can be detected by 2D gel and visualized by electron microscopy [89,90,91,92]. On the other hand, lacking the nucleases only results in persistent JM accumulation when STR is also mutated [92]. Several recent studies have expanded our understanding of how STR and JM nucleases are regulated to adhere to the temporal requirements of their activities.

### 5.1. Dissolution by STR Is Regulated by SUMOylation

Early observations that mutants of the SUMO E2 Ubc9, the Smc5/6 complex, and its SUMO E3 subunit Mms21 accumulate JMs when cells replicate in the presence of MMS suggest a role of SUMOylation in JM processing [38,71,74,93]. A simple interpretation would be that the Smc5/6 complex regulates the JM dissolution function of STR through SUMOylation. However, the negative genetic interactions between mutants of Smc5/6 and STR or their homologs in yeasts and mammals challenge this interpretation (e.g., [74,94]). Two recent studies address this issue and found that STR and Smc5/6 indeed show physical interaction upon replication stress [95,96]. As mutation of SIMs on Sgs1 or the Mms21 SUMO ligase function abrogate this interaction, the interaction is likely mediated by SIMs of Sgs1 and the SUMO modules on the Smc5/6 complex, which are conjugated largely by Mms21 [70,95,96,97]. They also show that STR-Smc5/6 association promotes Mms21-dependent SUMOylation of the STR complex [95,96]. As mutants defective for the Smc5/6-STR interaction or STR SUMOylation accumulate JMs and sensitize mutants of JM nucleases, STR SUMOylation is critical for efficient JM dissolution [95,96] (Figure 4). The mechanism of this effect pertains to SUMO-based recruitment of STR complex to repair foci or chromatin [95,96]. As recent studies show that Top3-Rmi1 also have the ability to process joint DNA structures independently of Sgs1 [98,99,100,101], it will also be interesting to understand how this activity contributes to JM processing during replication and whether it is regulated by SUMOylation.

### 5.2. Resolution Nucleases Are Regulated by Phosphorylation

Another set of studies showed that phosphorylation, rather than SUMOylation, regulates both the Mus81-Mms4 and the Yen1 nucleases [102]. For example, Mms4 phosphorylation in the G2/M phase depends on the mitotic cell cycle kinases Cdc5 and CDK and leads to increased nuclease activity [103,104] (Figure 4). Consistent with a role in JM processing, Mms4 phosphorylation is required for conferring genotoxic resistance and lowering JM levels in *sgs1* mutants [103,105]. On the other hand, the Mec1-Ddc2 checkpoint kinase complex limits Mus81-Mms4 activity by reducing the activities of CDK and Cdc5 [105]. Currently how phosphorylation of Mms4 promotes the nuclease activity is unclear, though this appears to involve additional factors that form a large complex.

These additional factors include three scaffolding proteins, namely Slx4, Dpb11, and Rtt107. Upon phosphorylation by Cdc5 in the G2/M phase, Mus81-Mms4 can interact with Dpb11 [106]. Phosphorylation of Slx4 by Cdk1 and Mec1 further boosts Dpb11 interaction [106,107]. Mutation of a major CDK phosphorylation site on Slx4 causes the persistence of JMs and increased MMS sensitivity in *sgs1Δ* cells, suggesting that the Slx4-Dpb11 complex contributes to JM resolution [106]. As this *slx4* mutant does not appear to affect Mus81-Mms4 nuclease activity [106], the role of the scaffold proteins might be targeting the nuclease to JMs or counteracting a checkpoint-mediated inhibition of the nuclease as described above. A detailed summary of how several kinases and scaffold proteins interplay in regulating resolution has been recently described [108,109]. 

Efficient Mus81-Mms4 activity also appears to require Srs2, which binds to and directly stimulates Mus81-Mms4 nuclease activity on 3’flap structures in vitro [110]. Consistent with this biochemical finding, the nuclease and Srs2 colocalize in cells with DNA damage [110]. It will be interesting to understand the timing of this interaction during the cell cycle and how this is connected to the phosphorylation and scaffold-mediated regulation of the nuclease.

## 6. Roles of Chromatin in Recombinational Repair

### 6.1. DNA Bending by Hmo1 Promotes Template Switching

Chromosome organization and chromatin states are known to influence homologous recombination processes [10,111]. Recent studies have examined the effects of chromatin organization factors in replication-coupled recombinational repair. One study examined the role of the high mobility group box (HMGB) protein Hmo1. Hmo1 and its homologs exhibit DNA bending, bridging, and looping activities in vitro and prefer binding to ssDNA or DNA with altered conformations, such as hemicatenane and cruciform structures [112,113,114,115]. In vivo, Hmo1 acts as a linker histone to stabilize chromatin and is implicated in Top2-mediated topological changes on chromatin [116,117]. Hmo1 loss reduces JM levels and suppresses the MMS sensitivity caused by removing the Rad5-Ubc13-Mms2 pathway, suggesting that Hmo1 is involved in template switching and that its function can be toxic in the absence of this pathway [118]. As the C-terminal DNA bending domain of Hmo1 was responsible for these effects [118], Hmo1 may use DNA bending as a mechanism to influence chromatin states or stabilize early JM intermediates.

### 6.2. Cohesion Contributes to Efficient Recombinational Repair during Replication

As sister chromatids provide the most accurate donors for each other’s repair, keeping sister chromatids close to each other conceivably favors recombinational repair. This notion is supported by the observation that defects in cohesin, the ring-shaped protein complex that can keep sister chromatids in proximity, reduce JM levels in *sgs1Δ* cells [119]. A recent study further showed that such a defect can be locally rescued when sister chromatids are artificially linked by the use of tetramerized LacI that can bind to both sister chromatids through integrated LacO arrays, but not by a dimerized LacI that can only bind to the LacO sites on one chromatid [120]. This rescue suggests a direct role of sister chromatid cohesion in recombinational repair, rather than other cohesin-related functions, such as transcription. As seen in many other situations, too much of a good thing can often be problematic. In this case, persistent cohesin on DNA prevents efficient recombinational repair of DNA breaks [121,122]. Removal of cohesin is expected to allow full access for recombinational repair factors to the site of repair, and this principle may also apply to repair during replication, though this remains to be directly examined. Taken together, these studies suggest that while cohesion is required for repair involving sister chromatids, in excess, it can block efficient repair.

When examining the roles of other factors required for cohesion in recombinational repair, the findings are mixed. In particular, while removal of Ctf4, a cohesion establishment factor, impairs JM formation, this defect cannot be bypassed by artificial cohesion mediated by tetramerized LacI as described above [120]. This finding suggests that Ctf4 contributes to recombinational repair by a mechanism beyond cohesion. Ctf4 is an integral part of the replisome and can tether primase, as well as several other factors to the replisome [123,124,125]. As primase mutants are also defective in JM formation [120], Ctf4 could affect JM formation through primase. Given that both Ctf4 and primase mutants exhibit increased levels of regressed forks as visualized by electron microscopy [120], efficient priming is likely required for a range of activities during replication, particularly replication fork progression and preventing the need for replication fork regression.

## 7. Concluding Remarks

Recent studies have expanded our understanding of the different modes of recombinational repair that facilitate replication. PCNA, which travels with the replication machinery, serves as one means to initiate and modulate recombinational repair. Abundant evidence shows that proteins generating three forms of modifications on PCNA and the readers of these modifications play important roles in dictating the choices between recombinational repair and translesion synthesis. In addition, modulating how much PCNA is retained on chromatin by the Elg1-containing alternative RFC complex is important. Besides PCNA, a similar DNA clamp, the 9-1-1 complex, can promote recombinational repair using a mechanism distinct from its defined role in checkpoint signaling [126]. Other recombinational repair factors, like the Shu complex and the Rad51 paralogs in other organisms, make important contributions via specialized roles during replication. Moreover, multi-functional DNA helicases influence recombinational repair, in part by promoting replication fork regression. Through functional interactions with checkpoint kinases and SMC proteins, they link these important genome maintenance factors to recombinational repair. Finally, chromatin modulators, such as Hmo1 and cohesin and its regulators, can influence recombinational repair by generating favorable chromatin order. 

As exploration in this field continues, additional players will certainly be added to those described here, and studying how their functions are integrated will eventually generate a comprehensive understanding of how recombinational repair facilitates replication. In the meantime, addressing many questions regarding the functions of known factors as exemplified above will delineate detailed mechanisms of repair. Many additional questions are also worthy of consideration, and a few examples are given below. For example, it will be important to gain better understanding into the temporal regulation of the different pathways. Through the use of genetic methods that can limit protein function to a particular cell cycle stage, PCNA modification pathways were shown to be able to exert their repair effects in the G2/M phase [127,128]. Yet, other studies show that these modification enzymes, such as Rad5, are most abundant and localize to nuclear subdomains in the S phase [63]. In addition, it has been shown that Rad51 and Rad52 are recruited to replication forks during the S phase, but the actual repair occurs in the G2/M phase [129]. Strategies to provide further temporal resolution of the functions of these proteins during different cell cycle stages will help to construct a cascade of events linked to the cell cycle. Another question that has not been well addressed pertains to observations that recombinational repair appears not to be error-free for all sites in the genome and can in fact be quite deleterious for repetitive sequences and heterochromatic regions [130,131]. Deeper insights into this aspect of error generation will benefit our knowledge of the repair outcomes of different regions of the genome. 

As Rad51 is central to recombinational repair, it is important to understand the additional roles of this protein and its interactors in other aspects of replication. For example, in human cells, Rad51 and its mediator proteins, such as BRCA2, can protect replication forks from nuclease degradation [132,133,134,135]. A similar role has been ascribed to several additional proteins in mammalian cells, including WRNIP1, PARP1, RECQ1, and BOD1L [19,136,137,138]. Whether yeast possess similar mechanisms remains to be understood. Moreover, Rad51 seems to have roles that can be harmful to replication, such as forming R-loop structures [139], which can lead to fork stalling, or binding to undamaged chromatin [140,141]. The latter can cause harmful effects, such as chromosome loss and growth arrest, and is counteracted by the Rad54 family proteins [140,141]. Additional layers of regulation likely are needed for balancing these diverse effects of Rad51. For example, phosphorylation has been shown to regulate Rad51 functions in both yeast and human cells [142,143]. Particularly, Nek1-mediated phosphorylation events promote Rad51 removal from DNA, and this regulation is confined to the G2 phase, favoring Rad51’s role in fork protection during the S phase [144]. In addition, the scaffold TopBP1, a homolog of Dpb11, promotes Rad51 phosphorylation by PLK1 to influence its function in recombination [143,145]. Clarifying the various modes of Rad51 regulation, in conjunction with further understanding the many other factors involved in replication-associated recombinational repair as described above, will enrich our understanding of how recombination can facilitate genome duplication and stability. This knowledge will be crucial for our understanding of the mechanisms underlying the many diseases associated with genome instability and designing new methods for their detection, prevention, and treatment.
